# Hemicelluloses/montmorillonite hybrid films with improved mechanical and barrier properties

**DOI:** 10.1038/srep16405

**Published:** 2015-11-09

**Authors:** Ge-Gu Chen, Xian-Ming Qi, Ming-Peng Li, Ying Guan, Jing Bian, Feng Peng, Chun-Li Yao, Run-Cang Sun

**Affiliations:** 1 Beijing Key Laboratory of Lignocellulosic Chemistry, College of Materials Science and Technology, Beijing Forestry University, Beijing 100083, China

## Abstract

A facile and environmentally friendly method was introduced to incorporate montmorillonite (MMT) as an inorganic phase into quaternized hemicelluloses (QH) for forming hemicellulose-based films. Two fillers, polyvinyl alcohol (PVA) and chitin nanowhiskers (NCH), were added into the hemicelluloses/MMT hybrid matrices to prepare hybrid films, respectively. The hybrid films were nanocomposites with nacre-like structure and multifunctional characteristics including higher strength and good oxygen barrier properties via the electrostatic and hydrogen bonding interactions. The addition of PVA and NCH could induce changes in surface topography, and effectively enhance mechanical strength, thermal stability, transparency, and oxygen barrier properties. The tensile strengths of the composite films F_PVA(0.3)_, F_PVA(0.5)_, and F_NCH(0.8)_ were 53.7, 46.3, and 50.1 MPa, respectively, which were 171%, 134%, and 153% larger than the F_QH-MMT_ film (19.8 MPa). The tensile strength, and oxygen transmission rate of QH-MMT-PVA film were better than those of quaternized hemicelluloses/MMT films. Thus, the proper filler is very important for the strength of the hybrid film. These results provide insights into the understanding of the structural relationships of hemicellulose-based composite films in coating and packaging application for the future.

The incorporation of layered silicates into polymers was first reported over forty years. Yet the serious exploitation of nanostructured clay composites began in the early 1990. A ground-breaking achievement was found by Toyota researchers in the field of polymer composites^1^,^2^. Since then, the field of clay nanocomposites has grown dramatically and inspired the development of a new class of materials. Earlier nanocomposites were obtained based on petroleum derived polymers, but in recent years, much attention was diverted to the use of natural polymers such as polysaccharides, polypeptides. Biodegradable materials have been a matter of research because of their abundance, low toxicity, biodegradability, and biocompatibility, which are used to supply the needs of society in the coming decades^3^,^4^,^5^. The natural polymer nanocomposites was based on a smaller volume fraction of layered silicates (clay), it can be used as a great potential for producing materials with the improved barrier, mechanical, and heat resistance properties^6^.

There were also some literatures on these natural biomass-based clay nanocomposite materials, such as thermoplastic starch^7^,^8^, cellulose^9^, and gelatin^10^. However, researches on hemicelluloses, their derivatives and clay hybrids film are very limited. Hemicelluloses represent about 20–35% of lignocellulosic biomass. Research has been developed recently because of the film-forming properties of hemicelluloses as well as biocompatibility and biodegradability^11^. Hemicellulose-based films are known to have good gas barrier properties due to their ability to form a dense macromolecular network with low mobility, and their excellent oxygen barrier properties make them of interest to packaging film applications^12^. However, the film based on hemicelluloses is proved to be poor mechanical strength and flexibility, as well as low moisture barriers^13^. These problems on the film of hemicelluloses are needed to be urgently dealt in future. Therefore, chemical modification had been applied to alter the characteristics in comparison to the parent compounds. It is reported that chemically modified hemicelluloses, such as cationic, carboxymethyl, and acylated hemicelluloses, have significantly enhanced the function of hemicelluloses^14^,^15^. It is also well known that the quaternization of hemicelluloses can enhance the solubility and yields of hemicelluloses, create novel opportunities to maximally exploit the various valuable properties of hemicelluloses for previously unperceived applications^16^,^17^. In addition, the additive is often necessary to ensure mechanical properties of hemicelluloses films, such as plasticizer and filler. In recent years, it is reported that the presence of MMT mixed into polymeric materials can reduce flammability and increase heat resistance at very low loadings (2–5 wt%)^18^,^19^. Montmorillonite, belongs to the family of 2:1 phyllosilicate, is capable of forming stable suspension in water. This hydrophilic character of MMT also promotes dispersion of this inorganic crystalline layer in water soluble polymers. It can be often used as reinforcing agent^20^,^21^. Moreover, the negative surface charge coming from the partial substitution of the trivalent Al-cation in the octahedral gibbsite layer by the divalent Mg-cation is balanced by sodium and calcium ions in the structure of MMT, which exist hydrated in the interlayer^22^,^23^,^24^. Guan *et al.* reported that the thermal and UV-vis transparency properties of hemicelluloses/MMT composite film were improved due to the proper proportion of hemicelluloses and MMT (1:1 v/v)^25^.

To improve the performance of quaternized hemicelluloses/MMT hybrid materials, some additives can be introduced as fillers to enhance the mechanical behavior. Polyvinyl alcohol (PVA) is a synthetic polymer with several nice properties, including non-toxicity, biocompatibility, high hydrophilicity, and film forming ability. It is particularly well-suited for the formulation of blends with natural polymers^26^. Chitin is the second most abundant biomacromolecule in nature. Native chitin in crab shells and squid-pens form crystalline fibrils of α- and β-chitin allomorphs. These α- and β-chitin fibrils have widths of 5–10 and 3–4 nm, respectively. Therefore, chitin has potential applications as bio-based fillers to reinforce polymer composites^27^,^28^. Increasing attentions have been focused on the engineered exploitation of chitin as biopolymer resources in many areas, including reinforcing nanocomposites, food industry, drug delivery, and tissue engineering^29^.

A number of articles regarding biomimetic ultrastrong composites have been successfully prepared by alternately deposition of clay sheets and polymers. However, this process is time consuming and only ultrathin films (<10 um) can be obtained within a reasonable time^30^,^31^. Based on the considerations, the first attempt to introduce a plasticizer (PVA or NCH) into quaternized hemicelluloses/MMT matrix, the nacre-mimetic layered quaternized hemicelluloses composite films with excellent mechanical and barrier properties were prepared using a simple, quick, and industrially scalable water-based processing approach akin to paper-making. Another major benefit of the processing approach is the “green” strategy, involving benign aqueous solvent, a minimum of up-scaled apparatuses, as well as readily available and biodegradable components. The hybrid films were prepared with quaternized hemicelluloses, MMT, PVA or NCH. The preparation and property studies of quaternized hemicelluloses with PVA and NCH nanocomposite film using this method have not been reported. In this study, low oxygen permeability as well as mechanical strength can be important target properties for quaternized hemicelluloses/MMT hybrid films. The morphological, thermal behavior, and mechanical properties of the composite films are further investigated by Fourier transform infrared spectrometer (FT-IR), X-ray diffraction (XRD), scanning electron microscopy (SEM), UV-vis spectroscopy (UV-vis), thermogravimetric analysis (TGA), oxygen transmission rate (OTR), atomic force microscopy (AFM), and tensile test.

## Results and Discussions

### Structural Analysis of Three Films

**Figure 1:**
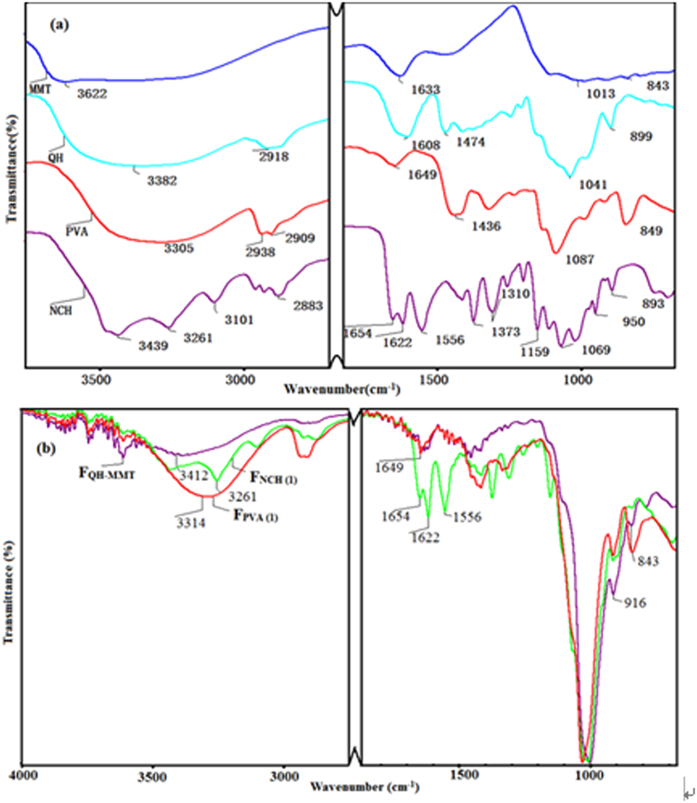
FT-IR spectra of MMT, QH, PVA and NCH (a). FT-IR spectra of the three types of composite films (F_QH-MMT_, F_NCH(1)_, F_PVA(1)_) (**b**).

FT-IR spectra of the QH, MMT, PVA, NCH are shown in Fig. 1a. As shown in Fig. 1a, the characteristic peaks of QH are presented at 3382, 2918, 1608, 1474, 1041, 899 cm^−1^. The absorptions at 3382 and 2918 cm^−1^ are indicative of the stretching of hydroxyl groups and the symmetric C-H vibration band, respectively. The band at 1608 cm^−1^ is originated from -COO^−^ of uronic acid and uronic carboxylate in hemicelluloses^32^. The band appearing at 1474 cm^−1^ is assigned to the CH_2_ bending mode and methyl groups of the substituent^33^. These peaks suggested that the quaternized hemicelluloses were prepared by etherification of hemicelluloses and ETA, introducing the cationic quaternary salt groups into the hemicelluloses macromolecules. The signal at 1041 cm^−1^ is attributed to C-O bond stretching frequencies. The band at 899 cm^−1^ is assigned to *β*-glycosidic linkages between the sugar units, indicating that the xylose residues forming the backbone of the macromolecule are linked by *β-*form bonds^34^. FT-IR spectra of the films, F_QH-MMT_, F_NCH(1)_, and F_PVA(1)_, are shown in Fig. 1b. The Al-OH stretching band at 3622 cm^−1^ and the Si-O-Si stretching band at 1013 cm^−1^ were observed at the MMT spectrum^35^. In the spectrum of F_QH-MMT_ film (Fig. 1b), the frequency of vibrational band at 3622 cm^−1^ is originated from Al-OH stretching band of MMT, and the peak at 843 cm^−1^ is assigned to the vibration of MMT. A peak appearing at 3382 cm^−1^ in the spectrum of QH shifted toward higher wavenumbers with addition of MMT, as was observed for example at 3412 cm^−1^ in the spectrum of the QH-MMT film. This shift of the OH stretching band was likely indicative of hydrogen-bonding interactions between QH and MMT^36^.

The spectrum of native chitin is shown in Fig. 1a. The peaks at 1654 cm^−1^ and 1622 cm^−1^ are assigned to the amide I region of α-chitin, the peak at 1556 cm^−1^ is attributed to the amide II region of α-chitin, and the peak at 1310 cm^−1^ is due to the amide III^37^,^38^. Another three peaks at 3439 cm^−1^, 3261 cm^−1^, and 3101 cm^−1^ are also observed, which represent the stretching of intramolecular and intermolecular OH and CH_2_OH vibrations, stretching of NH_2_, and NH secondary amides vibrations, respectively. These absorption peaks are especially consistence with the characteristics of pure α-chitin^39^. In the spectrum of the F_NCH(1)_ (Fig. 1b), the peaks at 1622 and 1556 cm^−1^ are assigned to the amide І region and the amide II region of chitin, indicating the occurrence of the molecular interactions among NCH, QH, and MMT. In addition, the peak at 3261 cm^−1^ which is assigned to the stretching of intermolecular OH, become stronger than that in the neat NCH. This was likely indicative of hydrogen-bonding interactions among NCH, QH and MMT. In Fig. 1a, the characteristic bands of PVA at 1087, 1436 cm^−1^ are assigned to stretching vibration of C-O-C and -CH_2_ bending, respectively. But in the spectrum of the F_PVA(1)_ film (Fig. 1b), the intensities of these two peaks become weak, which was attributed to the intercalation structure of F_PVA(1)_ composite film. Furthermore, the hydroxyl absorption band of the films (both F_NCH(1)_ and F_PVA(1)_) became stronger in the presence of NCH and PVA than the QH-MMT film, which indicated that the hydrogen-bond interactions were enhanced. These results also suggested that no chemical reaction was happened among QH, MMT, and NCH or PVA.

**Figure 2:**
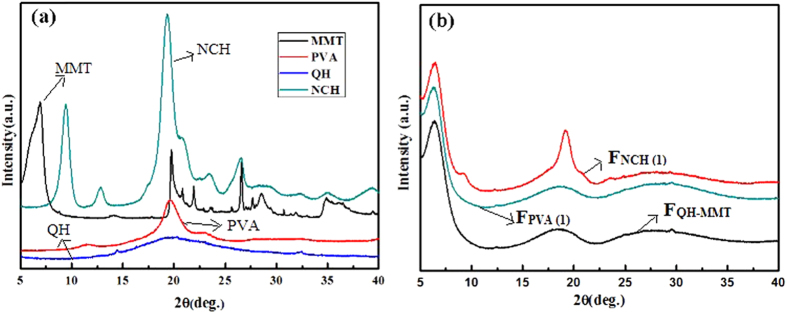
The XRD patterns of MMT, QH, PVA, NCH (a), and the three composite films (F_QH-MMT_, F_NCH(1)_, and F_PVA(1)_) (b).

In order to investigate the dispersion of the MMT layer in hemicelluloses matrix, X-ray diffraction analyses were performed. Figure 2(a) shows the XRD patterns of MMT, QH, PVA, and NCH. The characteristic peak of MMT was detected at 6.9° in the MMT pattern. It has been reported that the pristine MMT exhibited an amorphous X-ray diffraction pattern over Bragg angles of 3–9° 40. The XRD patterns of the three films are displayed in Fig. 2(b), all three diffraction peaks of MMT (6.3°, 6.4°, 6.2°) exhibited a lower angle compared with the original angle of MMT and became broad in the three films, which suggested that the interlayer spacing of MMT was increased because of the addition of filler. In the pattern of QH, only a broad peak can be observed at 19.9°. In Fig. 2(b) the diffraction peaks were shifted to 18.1° and 27.4° in the diffraction of the QH-MMT film. The primary peak of the PVA film is at 19.5°, and the diffraction peaks of the QH-MMT-PVA film were shifted to 18.7° and 28.5°. These results indicated that the intercalated hybrid structures were produced by electrostatic interaction and hydrogen bonding. The diffraction peaks of the composite films were shifted to higher angles, which was probably due to the change of crystal plane^41^,^42^. In other words, the deviation of angles was related to the decreases of the constant and volume of crystal lattice, and then the crystal plane was changed, which was mainly due to that the polymer was intercalated into the MMT nanoplatelets, and the new crystal plane was formed during this process. Five diffraction peaks of chitin powder were clearly exhibited at 2*θ* angles of 9.3°, 12.7°, 19.3°, 20.9°, 23.4°, and 26.2° in Fig. 2(a), which corresponded to the planes of (020), (021), (110), (120), (130), (013)^37^,^43^. Comparatively, in the pattern of the F_NCH(1)_ film (Fig. 2(b)), four diffraction peaks at 2*θ* angles of 9.3° (020), 19.3° (110), 20.9° (120), 23.4° (130), representing the crystalline structure of α-chitin, were still observed in F_NCH(1)_. The results indicated that α-chitin crystalline structure was maintained even after the incorporation of QH and MMT. This maybe the reason of the film F_NCH(1)_ exhibited much higher tensile strength and thermal behavior than that of the QH-MMT film, which need to be further discussed in the tensile and thermal behavior analysis. The characteristic peaks of the composite films with different kinds of fillers exhibited different intensities (Fig. 2(b)). As can be seen, the crystallinity of F_NCH(1)_ was lower than that of NCH, but the crystallization behavior of F_NCH(1)_ is better than other two films.

### UV-vis Transparency of Films

**Figure 3:**
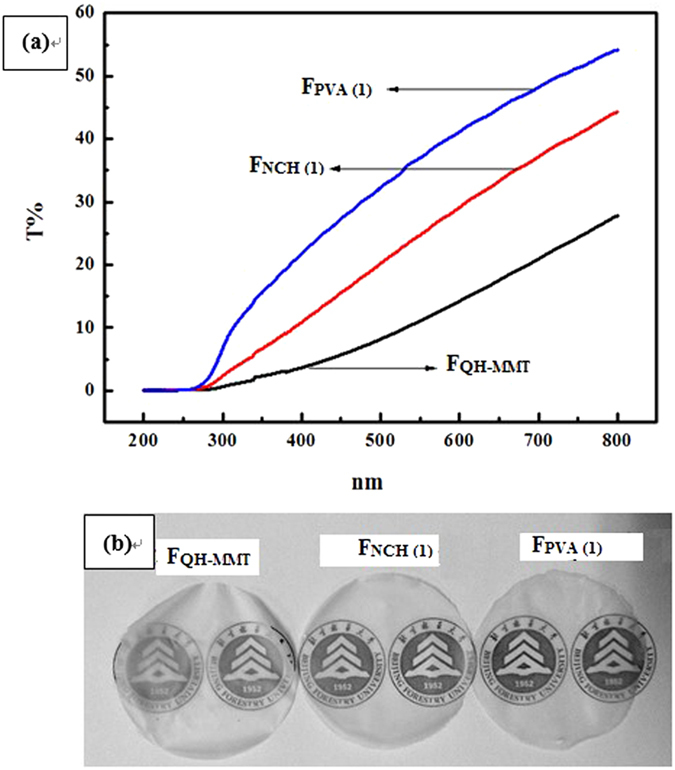
UV-vis spectra of the three composite films (a), the transmittance of the three types of composite films (b).

Figure 3(a) shows the light transmittance spectra of the films of F_QH-MMT_, F_NCH(1)_, and F_PVA(1)_. The transmission spectra of the three films in the UV light and visible light regions (200–800 nm) are affected by the presence of the clay loading in the composite films. As can be seen, the F_NCH(1)_ and F_PVA(1)_ films had higher transparency than F_QH-MMT_ film, which was due to the fact that the MMT content of the films was decreased. Among the three films, F_PVA(1)_ had much stronger transmittance than other two films (as shown in Fig. 3(b)), which suggested that the electrostatic interaction and hydrogen bonding among hemicelluloses, MMT, and PVA, were stronger than the interaction of the F_NCH(1)_ and F_QH-MMT_ film.

### Morphology of Films

**Figure 4:**
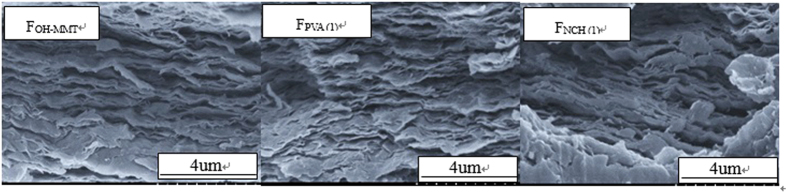
SEM photos of the fracture of the F_QH-MMT_, F_NCH(1)_, and F_PVA(1)_ films.

Assessment of the dispersion state of MMT in the QH and fillers matrix were studied by SEM, and this is essential to understand the composite behavior. The images of the cross-section of the three films presented a highly aligned structure in Fig. 4, indicating that every platelet was deposited in film lays flat. The orientation of MMT sheets in the film was possibly caused by directional flow induced by vacuum filtration and the electrostatic interaction and hydrogen bonding. MMT clay nanoplatelets were exfoliated to become negatively charged surface when they were immersed in water. The aqueous QH had positive charge. The electrostatic attraction between the two contrarily charged polymers is the driving force for the formation of multilayered films. This was conceptually similar to the hybrid materials fabricated by layer-by-layer assemble^44^. The nanoplatelet layered structure observed was obtained in a paper-making preparation process suitable for the large scale manufacturing. Multilayer formation can be attributed to the electrostatic interactions among MMT, QH and hydrogen-bonding interactions.

**Figure 5:**
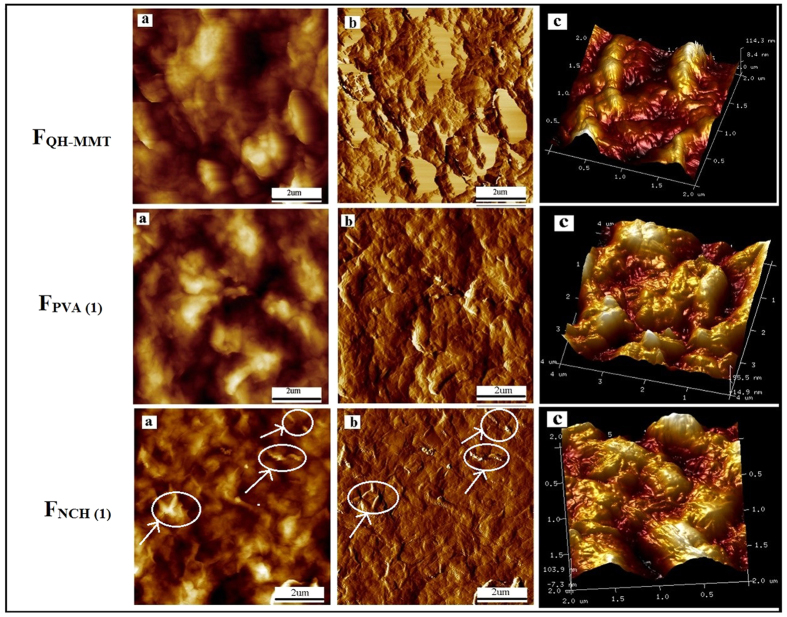
AFM height images, phase topography and 3D topography of the F_QH-MMT_, F_PVA (1)_, and F_NCH (1)_ films: F_QH-MMT_ (a–c); F_PVA(1)_ (a–c); F_NCH(1)_ (a–c).

The surface nanotopography of the three composite films were investigated by AFM. The height images, phase topography, and 3D topography of the films were studied. Figure 5 showed that the composite films exhibited a substantial rough surface as a result of the MMT platelets and fillers tightly connected with the QH macromolecular chain. The roughness values were calculated across 2 × 2 μm areas. The root-mean-square (RMS) roughness of the F_QH-MMT_, F_PVA(1)_, and F_NCH(1)_ were 98, 97, and 71 nm, respectively, which suggested that the nanoscale film layers were obtained. It was evident that most of the NCH were embedded in the hemicelluloses/MMT matrix, however, the randomly orientated rod-like of NCH were detected in the hemicelluloses/MMT matrices (Fig. 5). This may indicate that a NCH network began to form within the hemicelluloses/MMT matrix due to the rod-like morphology of NCH. The film F_NCH(1)_ was scanned at different locations and the profiles were similar, indicating that the obtained film F_NCH(1)_ presented a uniform surface of chitin nano-objects overlapping each other. The film F_NCH(1)_ presented lower RMS roughness due to their rod-like (nano-size fibrils) morphology, and NCH were in contact with each other leading to a continuous NCH network in the matrix. As showed in Fig. 5, MMT and the fillers can uniformly be dispersed in the hemicelluloses matrix. The presence of MMT contributed to the increase in the surface roughness. The surface roughness may play an important role in thermal behavior. During the electrostatic interaction, films with multilayers were formed through alternating sequential deposition of negatively and positively charged layers. That is to say, the hemicelluloses and fillers had been intercalated into the MMT nanoplatelets.

### Mechanical Properties of Composite Films

**Figure 6:**
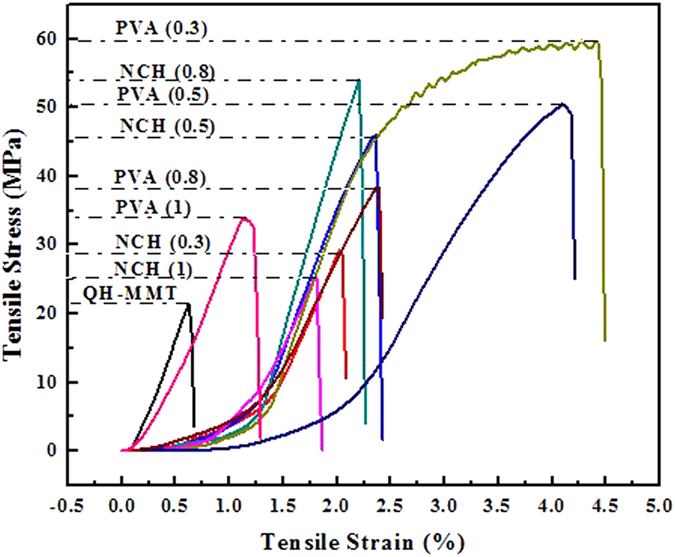
Tensile property for the composite films.

The effect of diverse additives on the mechanical properties of the composite films was studied in Fig. 6. The reinforcing effects of nanoparticle for polymers are related to the dispersion state of nanoparticles in the matrix and their interfacial interactions^1^. In addition, the composite films showed improved mechanical properties in virtue of the strong interfacial interactions among MMT, QH, and the fillers. As can be seen from Table 1, the tensile strengths of the composites films QH-MMT-PVA and QH-MMT-NCH were significantly improved owing to the addition of PVA and NCH, respectively. For example, the average tensile strength of F_PVA(0.3)_, F_PVA(0.5)_, F_NCH(0.8)_ are 53.7, 46.3, and 50.1 MPa, which were 171, 134, and 153% higher than the composite film F_QH-MMT_ (19.8 MPa), respectively. In addition, these values are better than or comparable to some reported films based on pure hemicelluloses (Table 1)^45^,^46^,^47^. For instance, films made from birch xylan birch had a tensile strength of 1.39 MPa^45^. The results are also comparable to the reported results from xylan films^46^,^47^. The elongation at break was remarkably increased for the film QH-MMT-NCH and QH-MMT-PVA compared with the F_QH-MMT_ film, indicating that the toughness of film F_QH-MMT_ is improved that could be ascribed to the high aspect ratio and strong interactions among NCH, PVA and the QH-MMT matrix. PVA is uncharged, nevertheless, it produces a stronger composite than do other polymers that undergo electrostatic attraction to the clay sheets^48^. This is explained by the high efficiency of hydrogen bonding and the higher viscosity of PVA, which result in better mechanical properties in QH-MMT-PVA film. This is also reasonable explanation that the higher concentrations give rise to thicker films, thus having a higher resistance toward deformation. Compared with other literatures^45^,^46^,^47^, films (QH-MMT-NCH and QH-MMT-PVA) were prepared from a lower concentration (1 wt %), and showed a higher strength. In other words, the degree of structural organization afforded by the process akin to paper making and the additive (PVA or NCH) were considered to be the result of improving the mechanical property of quaternized hemicelluloses/MMT film. For the biomacromolecule films (e.g., cellulose, starch) with or without external additives, mechanical strength mainly depends on the hydrogen bonds. External additives leads to a considerable decrease of strength due to the decreasing hydrogen bonds^49^,^50^. In other words, the additives can improve the film-forming performance of biopolymer at the cost of decreasing their tensile strength. In this work, quaternized hemicelluloses films without external additives cannot be prepared because too strong hydrogen bonds caused strong internal stress, resulting in cracks and defects within the film^51^. The presence of external additives (MMT, PVA and NCH) can produce a continuous film by replacing hydrogen bonds of hemicelluloses.

### Thermal Behavior Analysis

**Figure 7:**
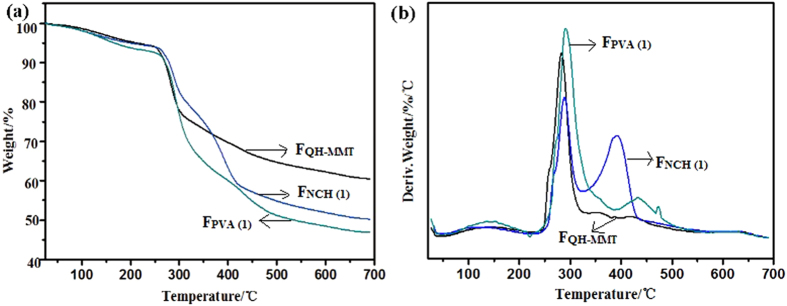
TGA and DTA curves of the three types of composite films.

The thermal stabilities of the F_QH-MMT_, F_PVA(1)_, and F_NCH(1)_ films were analyzed by thermogravimetric analysis (TGA), and the results were shown in Fig. 7. In the TGA curves, the decomposition process of the three films showed the first mass loss below 200 °C related to the volatilization of water. The rate of decomposition and weight loss of the three films was maximum at temperature of 200–500 °C, the primary cause was the degradation of polymers (hemicelluloses, NCH, and PVA), such as C-O band. In addition, the *T*_onset_ (the temperature at onset of the decomposition of polymer), *T*_1_ (the maximum weight loss temperatures), *T*_2_ (the lower maximum weight loss temperatures), and residual values of the composite films were determined from the DTG curves as shown in Table 2. Evidently, the *T*_onset_ of the film F_NCH(1)_ shift slightly toward the higher temperature (243.4 °C) than that of the F_QH-MMT_ (240.7 °C) and F_PVA(1)_ (232.6 °C), which is confirmed the enhancement of thermal stability of the addition of NCH. Furthermore, it is interesting to find that appearance of two distinct peaks (*T*_1_, *T*_2_) in the F_PVA(1)_ and F_NCH(1)_ film (Fig. 7b). The *T*_1_ of F_QH-MMT_, F_PVA(1)_, and F_NCH(1)_ were 283.1 °C, 291.1 °C, and 287.0 °C, respectively. The *T*_*2*_ of F_PVA(1)_ and F_NCH(1)_ film were 432.9 °C, and 391.9 °C, respectively. This implied that two crystalline phases such as a syndiotactic sequences and an atactic sequence may exist after incorporation of PVA or NCH into the hemicelluloses/MMT matrix^52^. After ~600 °C, the all curves became flat and mainly the inorganic residue (MMT) was remained. The amount of remaining solid residues at 700 °C of F_QH-MMT_, F_PVA(1)_, F_NCH(1)_ films were 60.2%, 46.5%, and 50.2%, respectively, implying that the thermal property of the composite film was improved with the intercalated structure, which was also due to the mobility of the biopolymer chains was decreased by the addition of MMT. In addition, as can be seen from Table 2, although the F_PVA(1)_ and F_NCH(1)_ films had the same amount of MMT, the F_NCH(1)_ had higher amount of remaining solid residues than that of F_PVA(1)_ at 700 °C. The reason was that the crystallization behavior of the F_NCH(1)_ was better than that of the F_PVA(1)_, and resulted in the relatively lower heat spread. This result is consistent with the XRD result (Fig. 2b).

### Oxygen Barrier Properties

Oxygen transmission rate (OTR, cm^3^/m^2^·24 h·0.1 MPa) of F_QH-MMT_, F_PVA(1)_, and F_NCH(1)_ were measured at 23 and 40 °C (0% RH). Experimental results on barrier properties are presented in Table 3. The OTR of F_QH-MMT_, F_PVA(1)_, and F_NCH(1)_ at 23 °C had relatively low value of 2.73, 1.93, and 1.37 cm^3^/m^2^·24 h·0.1 MPa, respectively. This phenomenon was ascribed to the inorganic phase (MMT), which introduced a physical barrier to diffusing molecules, increasing their tortuous path within the hemicelluloses and ultimately enlarging the characteristic length for the diffusive process^53^. The OTR of the three films (F_QH-MMT_, F_PVA(1)_, and F_NCH(1)_) at 40 °C was 12.26, 5.54, and 44.41 cm^3^/m^2^·24 h·0.1 MPa, respectively. Compared with the film F_QH-MMT_, the films F_PVA(1)_ and F_NCH(1)_ had relatively lower oxygen transmission rate at 23 °C. However, the OTR of film F_PVA(1)_ decreased significantly at 40 °C. In other words, F_PVA(1)_ film provided a remarkable OTR value at 23 and 40 °C (1.37, and 5.54 cm^3^/m^2^·24 h·0.1 MPa, respectively), which was a favorable value of high barrier coating used in the food packaging field. The OTR of film F_PVA_ may be enhanced due to the stronger hydrogen bonding in the film, resulting in the lower OTR value. In addition, the OTR of F_PVA(1)_ was lower than that of F_QH-MMT_, as expected the increased diffusion length was formed along the tortuous diffusion path in a nacre-like structure. The lowering of the penetrant mobility in the films F_PVA(1)_ at 23 and 40 °C, was due to that the effects of the hydrogen bonding of films (QH-MMT-PVA) was stronger than films QH-MMT-NCH and QH-MMT. In other word, this study has demonstrated that quaternized hemicelluloses/MMT films, in combination with PVA, can be profitably used generate oxygen barrier hybrid films.

## Materials and Methods

### Materials

The clay was a sodium montmorillonite with a cation-exchange capacity (CEC) of 90 mequiv./100 g and density *ρ* = 2.32 g·cm^−3^, originated from Alfa Aesar (China) Chemical Co., Ltd. The sugar composition of the hemicelluloses is: 83.5% xylose, 5.1% arabinose, 4.2% glucose, 0.4% galactose, and 6.8% glucuronic acid (relatively molar percent). The hemicelluloses had a weight average molecular-weight (*M*_*w*_) of 13 420 g·mol^−1^ with a polydispersity of 4.1, which was determined by gel permeation chromatography (GPC)^54^, corresponding to a degree of polymerization of 88. 2,3-Epoxypropyltrimethyl ammonium chloride (ETA) was obtained from sigma-aldrich Co., USA. Filter membrane, with 0.45 μm average pore diameter, is a microfiltration membrane which is made up of polyvinylidene fluoride (Jinteng, Tianjin). PVA were acquired from Beijing Yili Fine Chemicals Co., Ltd., China, and the degree of polymerization of PVA was 1750 ± 50. *α*-Chitin of high molecular weight (M_*w*_ = 5.0 × 10^5^ g·mol^−1^) was obtained from Zhejiang Golden-Shell Biochemical Co., Ltd., China. Chitin nanowhiskers were produced successfully using a method similar to the one described by Guan *et al.*^25^.

### Exfoliation of MMT

1.0 wt% MMT dispersion was prepared by stirring at 1000 rpm for 30 min, followed by sonication using a Scientz-II D (Ningbo Scientz Biotechnology CO. LTD) ultrasonic processor to allow swelling of the MMT clay. This process was repeated three times, and the solution was kept aside at room temperature for three days and subsequently centrifuged at 3800 rpm for 10 min to remove microbubbles and clay aggregates. The obtained supernatant MMT dispersion was applied for composite preparation.

### Preparation of Quaternized Hemicelluloses

Quaternized hemicelluloses were synthesized according to the literature described by Guan *et al.*^55^. Quaternized hemicelluloses were synthesized in three-necked flask fitted with a mechanical stirrer and a reflux condenser. Dry hemicelluloses (1.32 g) were suspended in 10 mL distilled water at 60 °C for 30 min with a speed of 400 rpm. An aqueous sodium hydroxide (the molar ratio of NaOH to ETA, 0.75) was added, followed by ETA (the molar ratio of ETA to anhydroxyloses units in hemicelluloses, 2.0). The mixed dispersions were stirred at 60 °C for 4 h and cooled to room temperature in order to ensure the completion of the reaction. Then the mixtures were thoroughly washed with ethanol and filtered off, dried in a vacuum oven at 60 °C for 24 h. The quaternized hemicelluloses had a weight average molecular weight (*M*_*w*_) of 9 240 g·mol^−1^, which was obtained by gel permeation chromatography (GPC). The DS_N_ of the modified hemicelluloses was 0.25, which was calculated from the ratio of the nitrogen to the carbon content according to the following equation: DS_N_ = (60×%N)/(14×%C-72×%N)^56^.

### Preparation of Composite Films

**Figure 8:**
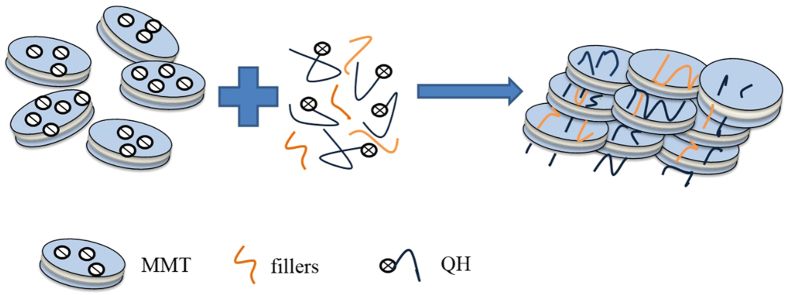
The forming mechanism of the composite film.

0.5 g PVA was dissolved in 50 mL distilled water at 90 °C with vigorous stirring for 30 min. 1 g of NCH was dissolved in 100 mL deionized water under mechanical stirring, then a 1 wt% NCH suspension was prepared. The concentrations of QH, MMT, PVA, and NCH were all 1%. QH-MMT composite film was produced via the intercalation of hemicelluloses into MMT. Firstly, QH-MMT dispersion (5 mL) was prepared with the proportion of 1:1 (v/v), and then the co-dispersion was subjected to vigorous stirring for 12 h before use. Secondly, the mixtures were vacuum-filtrated using a filter membrane for 20 min. The film QH MMT-NCH were prepared by mixing the suspension of QH, MMT, and NCH solution with the different proportion (1:1:0.3, 1:1:0.5, 1:1:0.8, and 1:1:1 (v/v/v)), and then the mixed solution was stirred vigorously at room temperature for 12 h, and then the solution (5 mL) was vacuum-filtrated with the filter membrane for 20 min. Finally, the wet films were vacuum-dried at 80 °C for 10–15 min and obtained by carefully peering off from the filtration membrane. The film QH-MMT-PVA was prepared as the same as the method of film QH-MMT-NCH. The composite films prepared from QH, MMT, and the additives are listed in Table 4. The forming process of the composite films is presented in Fig. 8.

### FT-IR Analysis

FT-IR spectra of the films were measured on a Thermo Scientific Nicolet In 10 FT-IR Microscope (Thermo Nicolet Corporation, Madison, WI) equipped with a liquid nitrogen cooled MCT detector. Dried samples were recorded with BaF_2_ disks in the range from 4000 to 650 cm^−1^ at a resolution of 4 cm^−1^ and 128 scans per sample.

### UV-vis Spectroscopy (UV-vis)

UV-*vis* spectra of sample films were collected on an ultraviolet/visible spectrophotometer (Tech comp, UV 2300) within the range of 200–800 nm. Before UV-vis measurements, the films were pasted on the surface of quartz pool.

### Atomic Force Microscopy

Thin film topography was carried out via a Multimode 8 Atomic Force Microscope (AFM) (Bruker, Germany). In the AFM scanning, two to four interest locations on each sample were tested. Small pieces of film samples were glued onto metal disks and attached to a magnetic sample holder located on the top of the scanner tube. Topographic (height) and phase images were collected in the tapping mode under ambient air conditions using a monolithic silicon tip with a resonance frequency between 250 and 300 kHz, and a scan angle of 0°.

### Scanning Electron Microscopy

The morphology of film samples was investigated by field emission scanning electron microscopy using a Hitachi S-3400N II (Hitachi, Japan) instrument at 15 kV. Prior to SEM observation, the specimen samples were sputter-coated with a thin layer of gold. Images were obtained at magnifications ranging from 200× to 5000×, which was dependent on the feature to be traced.

### Mechanical Property and Coating Thickness

The tensile tests of the films were performed with a universal materials testing machine (UTM6503, Shenzhen Suns Technology Stock CO. LTD. China). Specimens of 20 mm length and 20–30 um thickness and 10 mm width were tested with strain rate of 5 mm/min. The relative humidity was kept at 50% and the temperature at 23 °C. The thickness determination of specimens were performed with a paper thickness gauge (ZH-4, Changchun paper testing machine CO. LTD. China). The display of paper thickness gauge provides accurate reading with 0.001 resolution. The results for each specimen are based on at least 3 specimens, if nothing else is mentioned.

### X-Ray Diffraction and Thermal Behavior

Diffractograms were recorded in reflection mode in the angular range of 5–40 ° (2*θ*) at a speed of 5 °·min^−1^. The measurement was done with an instrument (Bruke, Japan) with a Cu K*α* radiation source (λ = 0.154 nm) at 40 kV and current 35 mA. Thermal behavior of the three films was performed using thermogravimetric analysis (TGA) and derivative thermogravimetry (DTG) on a simultaneous thermal analyzer (DTG-60, Shimadzu) under a nitrogen atmosphere from 25 to 700 °C and with a heating rate of 20 °C·min^−1^.

### Oxygen Transmission Rate (OTR)

The permeability of the material to oxygen at different temperatures (23 and 40 °C), the relative humidity conditions (0% RH) was investigated using the OX2/230 permeability analyzer equipped with a coulometric oxygen sensor. Test specimens were free from shriveling, folds, pinholes, and the specimens should be uniform thickness. Film samples with thickness of 25 ± 5 um were mounted in an isolated diffusion cell and were subsequently surrounded by flowing nitrogen gas to remove sorbed oxygen from the samples. The effective permeation area of the test specimen was 1.131 cm^2^, the gas flow rate is generally set at 5 mL/min. The results were expressed as the oxygen transmission rate (OTR, milliliters per square meter per 24 h at 0.1 MPa), which had been indicated as the most suitable unit for heterogeneous packaging materials^57^.

## Conclusion

A simple and industrially scalable water-based processing approach akin to paper-making has been used to prepare flat, heat-resisting, and excellent gas barrier properties hemicellulose-based hybrid films with montmorillonite platelets as inorganic phase, and the additive (PVA and NCH) as reinforcing agent. The electrostatic attraction and hydrogen bonding interactions between quaternized hemicelluloses and MMT were confirmed. MMT were uniformly dispersed in the hemicelluloses matrix, despite with the attendance of the fillers. The nanoscale film layers were obtained, and the surface of the composite films were homogeneous and smooth. The incorporation of PVA and NCH can induce changes in surface topography of QH-MMT composite films. The tensile property of QH-MMT film was enhanced by PVA and NCH significantly. Compared with the QH-MMT film, the films F_PVA(0.3)_, F_PVA(0.5)_ and F_NCH(0.8)_ showed higher tensile strength, higher optical transparency, and better oxygen barrier properties at 23 °C. Additionally, the tensile strength and OTR of F_PVA(1)_ film were all better than other films, suggesting that the PVA was more beneficial to affect the desirable properties of the composite films. Based on the above results, the addition of PVA and NCH can effectively enhance mechanical properties, thermal stability, transparency, and oxygen barrier properties. This hemicelluloses-based nanocomposite film can be a candidate material as a “green” and transparent replacement for non-renewable films, and still holds considerable promise for the future in packaging application.

## Additional Information

**How to cite this article**: Chen, G.-G. *et al.* Hemicelluloses/montmorillonite hybrid films with improved mechanical and barrier properties. *Sci. Rep.* **5**, 16405; doi: 10.1038/srep16405 (2015).

## Figures and Tables

**Table 1:** The results of tensile tests of the composite films.

Code	Thickness (um)	Tensile strength (MPa)	Tensile strain (%)	
F_QH-MMT_	24 ± 0.8	19.8 ± 1.4	0.5 ± 0.1	
F_PVA(0.3)_	23 ± 1.1	55.7 ± 5.9	3.9 ± 0.5	
F_PVA(0.5)_	26 ± 1.3	46.3 ± 5.4	4.0 ± 0.2	
F_PVA(0.8)_	26 ± 1.7	34.5 ± 3.8	2.3 ± 0.5	
F_PVA(1)_	28 ± 0.4	31.4 ± 2.3	1.1 ± 0.3	
F_NCH(0.3)_	25 ± 0.8	28.6 ± 1.6	2.3 ± 0.4	
F_NCH(0.5)_	23 ± 0.5	42.1 ± 3.8	2.1 ± 0.3	
F_NCH(0.8)_	25 ± 1.2	50.1 ± 4.2	2.2 ± 0.9	
F_NCH(1)_	22 ± 0.3	24.2 ± 1.9	1.7 ± 0.4	
**Films reported in literature**				
**reference**	**Major component**	**Thickness (um)**	**Tensile strength (MPa)**	**Tensile strain (%)**
45	xylan	290–380	1.08–1.39	45.5–56.7
46	Xylan-rich hemicelluloses and cellulose nanofibers	52–56	11.9–39.5	1.4–3.4
47	Long-chain succinic anhydride modified xylan	60–80	6.9–44	3.4–35.1

**Table 2:** Thermal characteristic of TG curves in Fig. 7.

Curve	*T*_*onset*_ (°C)	*T*_*1*_ (°C)	*T*_*2*_ (°C)	Residuals (wt%) at 700 °C
F_QH-MMT_	240.7	283.1	—	60.2%
F_NCH(1)_	243.4	287.0	391.9	50.2%
F_PVA(1)_	232.6	291.1	432.9	45.6%

**Table 3:** Oxygen transmission rate (OTR, cm^3^/m^2^·24 h·0.1 MPa) of the films F_QH-MMT_, F_PVA(1)_, and F_NCH(1)_.

Code	F_QH-MMT_	F_PVA (1)_	F_NCH (1)_
Thickness (um)	24 ± 0.8	28 ± 0.4	22 ± 0.3
23 °C 0%RH	2.73	1.93	1.37
40 °C 0%RH	12.26	5.54	44.41

**Table 4:** Different proportions of hemicelluloses, MMT, PVA or NCH in films.

Sample codes	Proportion
F_QH-MMT_	V(QH):V(MMT)=1:1
F_PVA(0.3)_	V(QH):V(MMT):V(PVA)=1:1:0.3
F_PVA(0.5)_	V(QH):V(MMT):V(PVA)=1:1:0.5
F_PVA(0.8)_	V(QH):V(MMT):V(PVA)=1:1:0.8
F_PVA(1)_	V(QH):V(MMT):V(PVA)=1:1:1
F_NCH(0.3)_	V(QH):V(MMT):V(NCH)=1:1:0.3
F_NCH(0.5)_	V(QH):V(MMT):V(NCH)=1:1:0.5
F_NCH(0.8)_	V(QH):V(MMT):V(NCH)=1:1:0.8
F_NCH(1)_	V(QH):V(MMT):V(NCH)=1:1:1
